# Depression in Fibromyalgia Patients May Require Low-Dose Naltrexone to Respond: A Case Report

**DOI:** 10.7759/cureus.22677

**Published:** 2022-02-28

**Authors:** Jagoda Siembida, Brian Johnson

**Affiliations:** 1 Psychiatry and Behavioral Sciences, State University of New York Upstate Medical University, Syracuse, USA; 2 Psychiatry, State University of New York Upstate Medical University, Syracuse, USA

**Keywords:** low-dose naltrexone, case report, hormonal system, chronic pain, fibromyalgia, depression, major depressive disorder

## Abstract

Fibromyalgia and depression are frequently comorbid. We propose a hormonal system model in understanding the underlying endogenous opioid system dysregulation in fibromyalgia with the utilization of the cold pressor test (CPT) in clinical practice to monitor treatment response to low-dose naltrexone (LDN) and the subsequent remission of major depressive disorder by restoring opioid tone.

A 60-year-old professional on permanent disability presented with refractory depression and chronic widespread pain after years of multiple failed medication trials. Rating scales confirmed severe depression, Hamilton Rating Scale for Depression (HAM-D) of 20, a short cold pressor test (CPT) time of 21 seconds, and a face pain scale (FPS) of 8/10. Physical examination assessing for fibromyalgia was diagnostic, with 18/18 positive tender points.

LDN, a minor increase in trazodone, and transference-focused psychotherapy were employed. The patient’s CPT time increased modestly. The patient achieved remission of both conditions in 10 weeks when both disorders were treated at once (FPS and HAM-D of zero), restoring the quality of life, relatedness, and motivation.

Some fibromyalgia patients may achieve remission of comorbid depression with concomitant low-dose naltrexone (LDN) treatment that is widely used “off label” to treat pain. LDN is a promising alternative for the treatment of chronic pain in fibromyalgia with its safety profile, high tolerability, and absence of abuse potential. Our unique finding is that without successful LDN treatment of fibromyalgia, remission of depression may be unlikely.

## Introduction

Anxiety disorders affect most patients with fibromyalgia [1], with a 40%-80% lifetime prevalence of depressive disorders [2]. Fibromyalgia presents with diffuse symptoms that include whole body pain, rapid transit of food through the gut often diagnosed as a separate disorder, “irritable bowel syndrome,” fatigue, sluggish thinking, “fibrofog,” and usually specific tender points that some physicians treat with “trigger point injections.” We have suggested [3-6] that fibromyalgia is a disorder of the opioid hormonal system that can be treated with low-dose naltrexone (LDN) to oppose an autoimmune attack on receptors. Momentary relief of pain with an opioid medication synergistically intensifies the underlying pathophysiology, as excess exogenous opioids result in the downregulation of mu-opioid receptors [6]. The overall response to opioids is known as the “opponent process.” Pain drivers overshoot an opioid blockade as if pain/tissue protection is necessary for life. Thus, chronic opioid therapy results in hyperalgesia [6]. The likely mechanism of naltrexone’s repair of opioid receptors is the transient blockade of the opioid growth factor receptor, causing the upregulation of the opioid growth factor [7].

Evidence suggests that the fibromyalgia brain is similarly dysregulated as the brain of the individual with opioid withdrawal. Both result in low opioid “tone” - the combined effect of circulating endogenous morphine and opioid receptors [3-6]. The endogenous opioid system is also a regulator of human interactions. Low opioid tone present in fibromyalgia [6] and high opioid tone present in some forms of autism [8] interfere with relatedness. Lack of relatedness is a driver of depression [9]. We suggest that because the opioid system is disrupted by the autoimmune attack on receptors, some patients with fibromyalgia cannot “feel” those they are closely related to, and perhaps also, they cannot feel their psychotherapist. The “feeling” of the patient is observed in the transference relationship, and the countertransference experience of the therapist is described in the case report.

We use the time of the cold pressor test (CPT), an ice water bath with a circulating pump, into which the patient submerges their normal forearm, as a proxy for central nervous system opioid tone [5]. Since the pain-damping opioid receptor system is impaired, patients with fibromyalgia have low pain tolerance as shown by short cold pressor test times [3-6].

We use the single case method to illustrate our clinical experience that depressive disorders may require fibromyalgia to be treated with low-dose naltrexone before the depression goes into remission. This is the constant experience on a neuropsychoanalytic service that is well documented with this particular case.

## Case presentation

Patient information

A 60-year-old male with chronic pain and refractory depression presented to our outpatient pain service with worsening depressed mood and anxiety. The patient’s daily functioning was impaired as he was unable to maintain an active lifestyle due to both pain and depression. The patient stated that his fibromyalgia, initially diagnosed eight years ago, flared up 2-3 times a month for about seven days at a time. He complained of burning and shooting pain radiating down the bilateral upper and lower extremities in addition to right-sided weakness. His pain was exacerbated by cold weather, physical activity, and prolonged inactivity. Review of systems was positive for fatigue, insomnia, weakness, tremors, urinary frequency, and myalgias of the bilateral upper and lower extremities.

Past medical history was notable for type II diabetes mellitus, diabetic neuropathy, coronary artery disease, hypertension, gastroesophageal reflux disease, fibromyalgia, osteoarthritis, sciatica, bilateral rotator cuff tears, and cervical nerve impingement with disc herniation and fusion. Past psychiatric history included major depressive disorder and one prior inpatient hospitalization at the age of 17 for severe depression. The patient denied any history of suicide attempts. Substance use history was positive for alcohol use 2-3 times monthly. The patient was a former smoker for 30 years. Illicit opioid use history was negative. Family history was notable for depression in the patient’s brother and mother. The patient was a previously high functioning professional. However, he was unemployed at the time of the initial intake and on permanent disability for completely refractory depression.

The patient had participated in psychotherapy and antidepressant treatment for most of his adult life, treatments ranging from weekly to twice weekly. Past medication trials for depression included SSRIs such as citalopram, escitalopram, fluoxetine, and norepinephrine dopamine reuptake inhibitor (NDRI) bupropion, all of which were poorly tolerated, and SNRIs venlafaxine and duloxetine. The patient’s first encounter with prescription opioids was approximately six years prior to presentation to our clinic. The patient suffered from C6-C7 ruptured discs and pain that severely impacted his ability to ambulate. He underwent a spinal procedure after injections failed to alleviate his pain. The patient was started on opioids shortly after the procedure and developed chronic constipation. His debilitating pain persisted despite treatment with “painkillers.” He stopped opioids five years before his presentation to our clinic. The patient also utilized benzodiazepines for over 15 years and clonazepam 0.5 mg three times daily and had never been off clonazepam for more than two weeks.

The patient’s presenting medication regimen included vortioxetine 20 mg daily, clonazepam 0.5 mg three times daily, trazodone 200 mg nightly, and gabapentin 800 mg three times daily (TID). Nonpsychiatric medications included aspirin, atorvastatin, clopidogrel, sodium docusate, famotidine, fluticasone, insulin, valsartan, and oxybutynin. He received cortisone injections for shoulder pain. The patient arrived at our clinic disabled and symptomatic, with severe depression and pain.

Clinical findings

Diagnostic Assessment

Our patient had the classic triad of generalized pain, 18/18 fibromyalgia tender points, and impaired CPT pain tolerance - understood as inability of the mu-opioid receptors to normally damp pain signals from the periphery. The diagnosis was consistent with persistent somatic symptom disorder, pain predominant, major depressive disorder, recurrent, severe, and fibromyalgia.

Therapeutic Intervention

Trazodone was raised to 300 mg for depression and insomnia, and gabapentin 800 mg was continued with a recommendation to take only at night. Reducing gabapentin dosing from TID to nightly improves both pain and insomnia. Low-dose naltrexone was initiated for fibromyalgia pain management. Dosing of low-dose naltrexone begins with 0.1 mg twice a day (BID) and is gradually increased over 11 days to a maximum of 4.5 mg twice a day. Patients may self-adjust dosing to control tolerability and response. Benzodiazepine discontinuation was strongly recommended. On our pain service, we give patients 10 chlordiazepoxide tablets 25 mg; put the decision on when to take them up to the patient; explain that, given the active metabolites, the functional half-life is five days; and explain that they will experience a blood level that they do not need to augment with any more chlordiazepoxide. Chlordiazepoxide self-taper properties worked perfectly, and the patient was off benzodiazepines in a week. There were no side effects of any medications.

As presented in Table 1, the patient’s HAM-D score of 20 was consistent with severe depression. The initial CPT time was 21 seconds in the context that 46 normal controls averaged 113 seconds [5]. The cognitive examination was normal. Screens for borderline personality and attention deficit hyperactivity disorder were negative. Physical examination included the evaluation of tender points for fibromyalgia; 18 out of 18 points were positive.

**Table 1:** Rating scales assessed at the time of the initial evaluation and 10-week follow-up HAM-D, Hamilton Depression Rating Scale; CPT, cold pressor test; FPS, face pain scale

Rating scale	Baseline	10-week follow-up
HAM-D	20	0
CPT	21 seconds	26 seconds
FPS	5/10, increased to 8/10 during evaluation	0/10

Follow-up and Outcomes

Our patient was scheduled for twice weekly follow-up for an extended evaluation using transference-focused psychotherapy [10]. Over the course of the 10-week follow-up period, with twice weekly 45 minutes of psychotherapy interrupted by a vacation taken in the middle of his treatment, the patient was initially noted to be significantly unrelated. During this period, the patient’s depression and fibromyalgia were symptomatic. He returned for follow-up visits with unremitting and debilitating depression. Vortioxetine 20 mg was deemed ineffective and tapered. Gabapentin was reduced to only 600 mg at bedtime. Table 2 summarizes our finalized medication changes leading up to remission.

**Table 2:** Psychotropic medication regimen at baseline versus at 10-week follow-up

Baseline	10-week follow-up
Vortioxetine 20 mg daily	Discontinued
Clonazepam 0.5 mg three times daily	Discontinued
Trazodone 200 mg nightly	Trazodone 300 mg nightly
Gabapentin 800 mg three times daily	Gabapentin 600 mg nightly

After 10 visits over 10 weeks, the patient’s symptoms resolved. Benzodiazepines had been discontinued, and the patient’s fibromyalgia was asymptomatic. The patient achieved remission of depression by the 10th session. He had an increase in goal-directed activity supplanting the previous amotivation and anhedonia. Our experience was that the unrelatedness was replaced by sparkling engagement and a sense of joie de vivre. The patient returned for repeat CPT, which improved to 26 seconds from 21 seconds, with a face pain score of 0/10 and HAM-D of 0, as presented in Table 1. At one-year follow-up, depression remained in remission and pain was minimal or absent. There were a total of 14 sessions with five, 13, 14, and 15 months follow-up notable for continued remission of depression and pain.

## Discussion

Depression and fibromyalgia, recognized as comorbid conditions by ample literature, are two distinct conditions leading to functional impairment and disability. Identifying these diagnoses with common overlapping symptoms may lead to a false conclusion that a single disorder is a symptom of the other. The understanding of fibromyalgia as a hormonal failure of the endogenous opioid system allows us to utilize low-dose naltrexone’s antagonistic actions, improving the individual’s opioid tone. Naltrexone improves the balance of the brain’s endogenous opioid system back toward normal. An additional mechanism of naltrexone’s therapeutic actions may involve decreasing the inflammatory response in fibromyalgia by antagonizing microglial toll-like receptors [11].

Low-dose naltrexone is a novel intervention for fibromyalgia with relatively few side effects. In a small double-blind, placebo-controlled, counterbalanced, crossover trial, the utilization of low-dose naltrexone reduced pain by 29% compared to placebo [12]. The same authors’ pilot study of 10 patients showed that low-dose naltrexone reduced fibromyalgia symptoms by greater than 30% over placebo assessed by mechanical and heat pain thresholds [13]. A small study of 12 patients in 2016 identified LDN as superior to placebo when treating major depressive disorder response with SSRIs and bupropion [14]. Low-dose naltrexone’s high tolerability vastly differs from FDA-approved first-line agents with significant side effects. Interestingly, the three agents, namely, pregabalin, duloxetine, and milnacipran, were shown to have either limited use due to the side effect profile or weak recommendations due to small benefits over placebo, without improvement in fatigue or quality of life [15]. A Cochrane Database Systematic Review in 2015 concluded that there is little evidence of treating the key symptoms of fibromyalgia with SSRIs compared to placebo [16]. These previous studies further support our hypothesis that the target treatment is improving low opioid tone to achieve remission of major depression and chronic pain. The current FDA-approved medications have no effect on the endogenous opioid system in contrast to naltrexone that we used off-label with success. Naltrexone was actually associated with reductions of the psychotropic regimen [17], as highlighted in our case by the discontinuation of an antidepressant and benzodiazepine and decrease in gabapentin dose while achieving remission of chronic pain and depression.

Literature review provides data that major depressive episodes are common in fibromyalgia patients [1]. Consistent with our model, pain and depression are independent of each other, as evidenced by a 2005 study of fibromyalgia patients using fMRI that investigated neuronal activations in separate brain regions involved in pain networks, a sensory component activated with painful stimulus and an affective component activated in patients with a diagnosis of major depressive disorder experiencing painful stimulus [18]. Depression was not correlated with the extent of the neuronal activation of the actual sensory component in the brain [18]. In our case, treatment with LDN explains the remission of both major depressive disorder and fibromyalgia as it targets the underlying pathophysiology of hormonal opioid imbalance.

It may be useful to compare depression in fibromyalgia with depression in hypothyroidism, an endocrine disorder presenting with major depression in some patients. A hormonal system is defined as a peptide that circulates through the blood with multiple tissue targets, such as thyroid hormone. Hypothyroidism presenting with major depressive disorder requires treatment of the hormonal disorder before the depression responds. At times, depression caused by hypothyroidism is cured by the provision of exogenous thyroid hormone. More commonly, the classic combination of psychotherapy and antidepressants will not cause a response to depressive symptoms unless thyroid function is corrected.

Since in this case, we treated both depression and fibromyalgia simultaneously, we cannot answer the question as to whether the response is a product of fibromyalgia treatment with low-dose naltrexone or the combination of increasing the dose of trazodone from 200 to 300 mg, shifting gabapentin dosing to only at bedtime with an ultimate reduction in dose to 600 mg, stopping the clonazepam 1.5 mg/day that was likely just dulling the patient, or discussing relatedness as a driver of depression, including observing it in the transference relationship.

The cold pressor test (CPT) is an objective test of pain that can be used to follow the effects of interventions for centrally mediated pain. The utilization of the CPT in clinical practice is useful to monitor treatment response. In a 2018 study, female controls achieved a CPT time of 111 seconds, and male controls achieved a CPT time of 114 seconds [5]. In comparison to our study, on initial presentation, the patient had a CPT time of 21 seconds. With the improvement of the endogenous opioid tone, hyperalgesia lessens and the CPT time increases. Low-dose naltrexone has few adverse effects, a safe profile, and high tolerability, with no abuse potential [19]. The effect size for low-dose naltrexone is 0.63 in fibromyalgia [6]. CPT times are substantially higher over two months of naltrexone 4.5 mg twice a day [3-6], usually accompanied by reports of decreased diffuse body pain.

The patient’s less robust increase in CPT may have several explanations, some unknown, although we suggest careful consideration of confounding factors present, which contributes to the limitations of this case report.

Because single-subject research includes multiple assessments, an individual can serve as her or his control over time [20]. However, a larger reproducible study is needed. We recommend and plan larger, randomized, placebo-controlled, double-blinded clinical trials to investigate low-dose naltrexone treatment and its effects on primary and secondary outcomes.

## Conclusions

Our case highlights the potential importance of using LDN in fibromyalgia patients with comorbid depression. Symptoms of pain, depression, and functional impairment may be reduced or put into remission with improvement in human interactions - a marker of increased opioid tone. 

To say it another way, humans are social animals. We fall ill when not related. Depression is one disorder caused by unrelatedness. Fibromyalgia causes unrelatedness by impairing opioid tone. Without fixing the autoimmune unrelatedness with low-dose naltrexone, in some cases, it may be impossible to put depression into remission.
